# Nutritional Composition and Bioactive Components in Quinoa (*Chenopodium quinoa* Willd.) Greens: A Review

**DOI:** 10.3390/nu14030558

**Published:** 2022-01-27

**Authors:** Safiullah Pathan, Rafat A. Siddiqui

**Affiliations:** 1 Department of Agriculture and Environmental Sciences, Lincoln University of Missouri, Jefferson City, MO 65101, USA; 2 Agricultural Research Station, Virginia State University, Petersburg, VA 23806, USA; rsiddiqui@vsu.edu

**Keywords:** quinoa, nutrients, bioactive components, greens, microgreens, sprouts, leafy vegetable, health benefits

## Abstract

Quinoa (*Chenopodium quinoa* Willd.) is a nutrient-rich grain native to South America and eaten worldwide as a healthy food, sometimes even referred to as a ”superfood”. Like quinoa grains, quinoa greens (green leaves, sprouts, and microgreens) are also rich in nutrients and have health promoting properties such as being antimicrobial, anticancer, antidiabetic, antioxidant, antiobesity, and cardio-beneficial. Quinoa greens are gluten-free and provide an excellent source of protein, amino acids, essential minerals, and omega-3 fatty acids. Quinoa greens represent a promising value-added vegetable that could resolve malnutrition problems and contribute to food and nutritional security. The greens can be grown year-round (in the field, high tunnel, and greenhouse) and have short growth durations. In addition, quinoa is salt-, drought-, and cold-tolerant and requires little fertilizer and water to grow. Nevertheless, consumption of quinoa greens as leafy vegetables is uncommon. To date, only a few researchers have investigated the nutritional properties, phytochemical composition, and human health benefits of quinoa greens. We undertook a comprehensive review of the literature on quinoa greens to explore their nutritional and functional significance to human health and to bring awareness to their use in human diets.

## 1. Introduction

Quinoa (*Chenopodium quinoa* Willd.) is a summer annual dicotyledonous herbaceous crop of the Amaranthaceae family. It was domesticated first in the Andean countries of South America about 7000 years ago. The Andean people began the cultivation of quinoa, possibly for its nutritional values, drought tolerance, and ability to grow in high salt conditions. The Incas considered quinoa a sacred crop and called it the ”mother grain”. Although overlooked for thousands of years, the agronomic and nutritional importance of this crop was rediscovered during the last 50 years, leading to a resurgence in its production. The number of countries growing quinoa has increased rapidly from 8 in 1980 to 40 in 2010 and to more than 100 countries in 2021. South American countries, namely Bolivia, Ecuador, and Peru, lead production, and these together account for more than 80% of the world’s production [1,2,3]. There are about 250 species of Chenopodium worldwide [4]. Quinoa grains are the main edible part, and gluten-free grains contain high quantities of protein, essential amino acids, and essential minerals and vitamins. Because of these nutritional properties and health benefits, quinoa is considered a novel healthy food, occasionally referred to as a ”superfood”. Considering its importance, the United Nations General Assembly declared 2013 as the ”International Year of Quinoa”.

**Figure 1:**
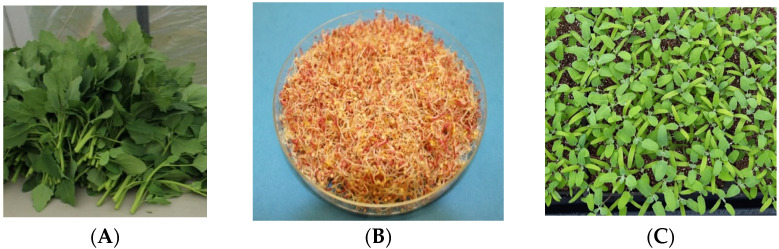
Representative pictures of quinoa greens, (**A**) green leaves, (**B**) sprouts, and (**C**) microgreens.

Like quinoa grains, quinoa greens (quinoa green leaves, sprouts, and microgreens; Figure 1) are rich in nutritional values and health promoting properties such as antimicrobial, anticancer, antidiabetic, antiobesity, antioxidant, and cardio-beneficial. The Incas consumed quinoa grains and leaves in their diet to balance the lack of animal proteins [5]. The people of the Andean regions have traditionally consumed quinoa leaves. The fresh leaves and tender shoots can be eaten as cooked vegetables and as a salad where quinoa sprouts can be added [6,7,8,9]. Researchers have recently reported that quinoa leaves contain high quantities of protein and all amino acids necessary for humans and low quantities of carbohydrates. Quinoa leaves also contain high levels of potassium, manganese, and copper and moderate levels of calcium, phosphorus, sodium, and zinc [10,11,12]. One of the species of genus *Chenopodium*, *Chenopodium album*, a close relative of quinoa, is a common weed known as ”lamb’s quarters”. *Chenopodium album* (*C. album*) originated in India and has been cultivated as a non-traditional vegetable in India and Bangladesh (Known as ”Bathua” in Hindi and Bengali). In the Indian subcontinent, young leaves and shoots of lamb’s quarters are eaten as vegetables. Although it is a weed, the young green leaves and tender stems are rich in different nutrients such as protein, fat, fiber, essential amino acids, minerals, and vitamins. They are rich in various bioactive compounds and have many medicinal properties for humans, such as antimicrobial, antihelmintic, antioxidant, antidiarrheal, and hepato-protective [13,14,15,16,17]. Phenotypically both *Chenopodium quinoa* and *C. album* appear very similar and hard to differentiate. Both are rich in nutritional and functional properties.

Many research and review articles have been published on the nutritional and bioactive components of quinoa grains [18,19,20,21]. However, only a few research articles on quinoa greens are available regarding their nutritional and phytochemical composition and human health benefits (Table 1). Furthermore, most research has been conducted in Europe and Asia, whereas only a few investigations have been carried out in the USA. Until now, no review article has been available covering this topic. To the best of our knowledge, this is the first review article covering the potential benefits of quinoa greens.

## 2. Nutritional Composition

A comparison of nutritional composition (proximate composition: protein, fiber, ash, and moisture; essential amino acids; and essential minerals) of quinoa (*Chenopodium quinoa*) greens (quinoa green leaves, sprouts), quinoa grains, and *C. album* leaves is presented in Table 2. The table was prepared based on available published articles. We noticed that data are variable. This may be due to the different locations and conditions under which the studies were conducted, the experimental variants used (different varieties, soil types, mineral composition, fertilizer application, and sampling time), and the analytical methods used.

### 2.1. Protein

The protein content of young (30–45 days old) dry leaves of quinoa ranged from 28.2 to 37.0 g 100 g^−1^ on a dry weight (DW) basis, and protein content of quinoa grains ranged from 9.1 to 15.7 g 100 g^−1^ (Table 2), indicating that dried leaves of quinoa contain a higher amount of protein than grains. Alternatively, Chacaliaza-Rodríguez et al. (2016) [22] used fresh leaf quinoa and reported that protein was low, about 4.6% at 86.4% moisture content. Quinoa sprouts contain a low amount of protein ranging from 6.1 to 12.3% [30,39]. Protein content in *C. album* dry and fresh leaf was about 28.7% and 5.0%, respectively [13,44]. Protein contents in both dry and fresh leaves of quinoa and *C. album* are comparable. Quinoa has a high biological value (the proportion of absorbed protein from a food which becomes incorporated into the proteins of the organism’s body) (73%), similar to that of beef (74%) and higher than wheat (49%) and corn (36%) [4,42]. The main proteins in quinoa are albumins (35%) and globulins (37%), whereas the prolamins are present in very low quantities [45].

Proteins of green leaves of quinoa are high in lysine (1.9 g), methionine (0.6 g), and threonine (1.5 g 100 g^−1^ protein) [12]; these are the limiting amino acids in conventional cereals, for example, wheat and maize [46].

### 2.2. Fat

The fat content of green leaves of quinoa ranged from 2.4 to 4.5%, lower than in quinoa grains (4.0 to 7.6%; Table 2). The fatty acid (FA) profile of quinoa plant parts at different growth stages compared with spinach, kale, and quinoa grains is presented in Table 3. Peiretti et al. (2013) [28] analyzed fatty acids and the nutritive value of quinoa grains and plant parts at different growth stages. At the early vegetative stage of quinoa, alpha-linolenic acid (ALA) was most abundant at about 47%, and linoleic acid (LA) was 16% of total fatty acid (TFA). In contrast, quinoa grain was characterized by a high amount of LA ranging from 46.69 to 58.10% and a low amount of ALA ranging from 6.10 to 8.44% of TFA [9,47]. It is clear that early and mid-vegetative growth stages of quinoa plants contain more ALA than LA. Both spinach and kale contain more ALA than LA [48,49], and results are comparable with the results of the quinoa early vegetative stage. The balance between omega-6 and omega-3 is critical in health risk reduction [50]. The above reports summarized that quinoa greens and early to mid-vegetative plants contain a significantly high amount of omega-3 (ALA); however, quinoa grains contain a high amount of omega-6 (LA). It is suggested that higher dietary intake of omega-3 fatty acids is associated with risk reduction in cardiovascular disease that may suppress inflammatory responses and benefit individuals with other chronic diseases.

### 2.3. Fiber

Based on solubility, there are two types of fiber in plants, soluble and insoluble. Pectin and gums are soluble in water (soluble fiber), whereas polysaccharides, cellulose, and hemicellulose are insoluble in water (insoluble fiber). Based on digestibility, fibers are divided into neutral detergent fiber (NDF) fractions containing hemicelluloses, cellulose, lignin, and acid detergent fiber (ADF) fractions containing mainly cellulose and some lignin. The amount of fiber present in quinoa leaves, sprouts, and grains ranged from 6.9 to 7.8, 4.6 to 23.5, and 7.0 to 14.1%, respectively (Table 2). In fiber fraction analysis of sprouted quinoa, Bhathal et al. (2017) [30] found a significantly higher amount of neutral detergent fiber (NDF), about 77.73%, compared to acid detergent fiber (ADF), only 11.63%. Peiretti et al. (2013) [28] reported 44.63 and 21.85% NDF and ADF, respectively, in the early vegetative stage of the quinoa plant. Quinoa grains are also an excellent source of dietary fiber with 78% insoluble and 22% soluble fiber [8,51,52]. A moderate level of fiber in quinoa leaves, which is close to spinach, contributes to the overall nutritional value [12].

### 2.4. Carbohydrate

The carbohydrate content was typically estimated using the following equation: carbohydrate (%) = 100% (protein + ash + fat + moisture). Total carbohydrate content in quinoa leaves (34.0%) was significantly lower than that of quinoa grain (48.5–69.8%), whereas 40.8% carbohydrate was reported in *C. album* leaves (lamb’s quarters) (Table 2). Starch is the major component of carbohydrates and comprises about 60% of dry weight [3]. Quinoa starch comprises 58.1% to 64.2% of dry seed weight with a low glycemic index [4]. The starch mainly constitutes D-xylose (120 mg 100 g^−1^) and maltose (101 mg 100 g^−1^) with low glucose (19 mg 100 g^−1^) and fructose (19.6 mg 100 g^−1^) content [6]. Foods containing high protein and low carbohydrates are beneficial to human health, as they do not contribute to raised plasma glucose.

### 2.5. Essential Amino Acids

Protein and amino acids (AA) are macromolecular compounds that have an essential role in the body as catalysts for structural components, enzymatic reactions, energy sources, and protein synthesis [53]. The nutritional quality of protein is determined by the concentration of essential amino acids that humans cannot synthesize in their bodies. Interestingly, all essential amino acids necessary for human growth and function are present in quinoa greens. The composition of essential AA of quinoa leaves, sprouts, grains, and leaves of *C. album* is presented in Table 2. The most abundant essential AA in quinoa leaves are leucine (2.7), lysine (1.9), phenylalanine (1.8), and valine (1.8 g 100 g^−1^ DW). In quinoa sprouts, the abundant AA are leucine (2.0), lysine (1.3), valine (1.3), and phenylalanine (1.2 g 100 g^−1^ DW). Lysine is deficient in many grains [42,54]. Methionine is generally deficient in green leaves but is found in higher levels in quinoa leaves than amaranth, spinach, moringa, and *C. album* leaves [12]. Methionine and cysteine are potent antioxidants that help in the detoxification of harmful components and protection from radiation [55].

### 2.6. Minerals

The composition of essential minerals of quinoa leaves, sprouts, grains, and leaves of *C. album* are presented in Table 2. The ash content is a measure of the total quantity of minerals present in a food. Ash contents in quinoa leaves ranged from 2.1 to 20.0%. Quinoa greens, especially quinoa leaves are rich in essential minerals including calcium (Ca), magnesium (Mg), phosphorus (P), potassium (K), iron (Fe), and zinc (Zn), and their richness compared with quinoa grains and *C. album* leaves is presented in Table 2. On a dry weight basis (mg 100 g^−1^), quinoa leaves range for Ca from 147.0 to 1535.0, Mg 14.0 to 902.0, P 39.0 to 405.6, K 474.0 to 8769.0, Fe 11.6 to 148.0, and Zn 3.3 to 6.8 mg 100 g^−1^. These values are comparable with the values obtained for the leaves of amaranth and spinach [12] and *C. album* [13,42]. Only two studies reported that quinoa sprouts are also rich in K and Mg, and the values are 525.0 and 219.0 mg 100 g^−1^, respectively [29,39].

### 2.7. Vitamins

There are few reports of vitamins quantification in quinoa greens. Among different vitamins, vitamin C (ascorbic acid) is an essential nutrient with a robust antioxidant capacity that the human body cannot synthesize and must be acquired from fruits and vegetables. The amount of vitamin C per 100 g of quinoa leaves ranged from 70 to 230 mg [6,11], 4-day-old sprouts ranged from 40 to 52 mg [56], and 20-day-old seedlings ranged from 37 to 70 mg 100 g^−1^ [29]. Quinoa leaves contain vitamin A (2085 mg) and a small amount of vitamin E (2.9 mg 100 g^−1^) [57]. Common leafy green spinach contains vitamin C ranging from 30 to 130 mg 100 g^−1^ [58,59]. However, quinoa grains contain 16 mg 100 g^−1^, whereas wheat, corn, and rice contain non-traceable amounts of vitamin C [60,61]. The above data suggest that quinoa greens are a good source of vitamin C.

## 3. Bioactive Compounds/Functional Compounds

Bioactive compounds include a variety of compounds present in small quantities in plants and plant-derived foods and provide health benefits beyond the nutritional value. It is suggested that intake of bioactive compounds rich food help in reducing oxidative stress and in prevention of cancer, heart diseases, and other diseases. Important bioactive compounds include polyphenols, carotenoids, tocols, phytoecdysteroids, phytosterols, bioactive proteins, and peptides as well as saponins, the most studied anti-nutrients, and other anti-nutrients including tannins and phytic acid.

### 3.1. Total Phenolic Content (TPC)

The TPC was measured in quinoa leaves and sprouts, mostly using the Folin–Ciocalteu assay, and data are expressed as mg gallic acid equivalent (GAE) per 100 g of sample dry weight basis (Table 4). The values ranged in the leaf from 131.80 to 544.00 and in the sprout from 49.02 to 417.75 mg GAE 100 g^−1^ DW, excluding some very high values [23,27,29]. The highest value of 544.00 was found in quinoa leaf [10], and the lowest of 79.04 mg GAE g^−1^ DW was found in quinoa sprout [32]. The TPC in quinoa grains ranged from 39.29 to 198.23 mg GAE 100 g^−1^ DW [31,32] (Table 4). During germination, biochemical, physical, and enzymatic activities lead to an increase in TPC in sprouts [62]. Different authors [33,63,64] have reported an increase in TPC during the seed germination process of quinoa. Hence, germination is used as a strategy to increase the quantity of bioactive compounds such as phenols, anthocyanins, and flavonoids [65,66].

The differences in the TPC as reported by different authors are due to growing conditions, soil type, as well as the variety, since the colored varieties have a higher TPC [9,31,34,67,68,69].

### 3.2. Total Flavonoid Content (TFC)

The TFC was measured in quinoa leaves and sprouts and expressed as mg quercetin equivalent (QE) per 100 g of sample dry weight basis (Table 4). The values ranged from 10.38 to 304.10 mg QE 100 g^−1^ DW, excluding some very high values [22,27,29]. The highest value of 304.10 was found in quinoa sprouts [35] and the lowest value in quinoa sprouts (10.38 mg QE 100 g^−1^ DW) [34]. The TFC in quinoa grains was about 11.40 to 223.80 mg QE 100 g^−1^ DW [36,61]. Flavonoids, such as rutin, quercetin, and kemferol, gradually increased with the progression of germination (9 days) and values were higher in yellow quinoa sprouts than red quinoa sprouts [34]. Exceptionally high TPC and TFC values were reported [22,23,27,29] for leaves and sprouts.

### 3.3. Antioxidants

Antioxidants are molecules that can prevent or reduce damage to cells caused by free radicals (known as reactive oxygen species, ROS), and plant-based foods are rich in antioxidants [70]. Different assays have been used to determine antioxidant capacity (AC) of quinoa greens, including chemical assays such as the 2,2-diphenyl-1-picrylhydrazyl (DPPH) method (Table 4). A comparison of results on quinoa greens obtained by different researchers is not always possible because of the use of different extraction methods, different assays for quantification, different varieties with different seed colors, samples from different plant parts, and growth stages used.

In general, radical scavenging activity measured in quinoa greens using DPPH was higher in infructescence (the fruiting stage of an inflorescence) followed by leaves and sprouts. Debski et al. (2018) [24] reported antioxidant activity in quinoa leaves, stems, and infructescence and compared with *C. album* and found similar trends in both the species. Alvarez-Jubete et al. (2010) [33] reported that the capacity of DPPH was comparable in quinoa sprouts and quinoa grains. Quinoa sprouts contain more antioxidant than quinoa grains and increase in antioxidant activity depends on the length of germination (up to 6–9 days) [34,36]. Red quinoa sprouts have a higher antioxidant capacity than yellow quinoa. Germinated quinoa grains (quinoa sprouts) revealed a considerable increase in antioxidant capacity due to physiological and biological changes during seed germination [63].

### 3.4. Carotenoids

Carotenoids are a group of pigments that are largely produced by plants and algae. Carotenoids play an important role in human health nutrition as precursors for vitamin A and as antioxidants. The most important and most studied carotenoids are beta-carotene, lycopene, and lutein. Bhargava et al. (2006) [6] evaluated 27 *Chenopodium quinoa* germplasm lines, and the average leaf carotenoid content was 48.41 mg, with a range from 23.02 to 66.96 mg 100 g^−1^ fresh weight (FW). Prakash et al. (1993) [71] reported a much lower quantity of total carotenoids in quinoa leaves in 10 germplasm lines, ranging from 8.20 to 19.00 mg 100 g^−1^. On the contrary, a higher quantity of carotenoids detected in leaves of two quinoa lines ranged from 64.24 to 90.50 mg 100 g^−1^ DW [22]. Total carotenoid content determined in sprouts of 6 days after germination in red and yellow quinoa grains was 15.58 and 8.11 mg 100 g^−1^, respectively [34]. Le at al. (2021) [29] reported that the amount of beta-carotene in 25-day old sprouts of 10 quinoa lines ranged from 12.42 to 32.71 mg 100 g^−1^ FW.

## 4. Antinutritional Factors

Molecules that react with nutrients, thus interfering with their absorption, are referred to as antinutrients [63]. Saponins, phytic acids, oxalates, tannins, and trypsin inhibitors are the main antinutritional elements found in quinoa.

### 4.1. Saponins

Saponins are the secondary metabolites, mostly found on the outer layer of grains, which provide bitter taste. Quinoa grains contain about 0.00–4.4% saponins [72,73]. In addition to grains, saponin contents have been also investigated in quinoa leaves, sprouts, stems, and bran [74,75,76]. Lim et al. (2017) [75] reported saponin contents in different parts of quinoa plants, such as leaves (0.97), grains (1.26), sprouts (1.29), stems (3.67), and bran (8.34%). Different factors such as locations, abiotic stresses, and varieties influence saponin contents. Environmental factors, such as drought and salinity, decrease the quantity of saponins, and the sweet quinoa variety has lower saponin contents than the bitter variety. In contrast to the cytotoxic effects of saponins, some health promoting properties of quinoa saponins have been reported such as anticancer, antiobesity, neutralizing free radicals, and reducing heart diseases [19,77].

### 4.2. Phytic Acid

An excessive amount of dietary phytate capable of chelating bivalent minerals, such as iron, calcium, magnesium and zinc reduces the absorption of these elements. The presence of a small amount of phytic acid in food can help in phosphorus storage. To the best of our knowledge, no report available on phytic acid amount in quinoa greens. Phytic acid contents in quinoa grains ranging from 200 to 880 mg 100 g^−1^ [78,79]. Phytic acid content in the leaves of several *C. album* varieties ranged from 238 to 268 mg 100 g^−1^ [80].

### 4.3. Oxalate

Oxalates are antinutrient components that can bind with Ca, Fe, and Mg ions and hinder availability of nutrients. High intake of soluble oxalate in diets decrease bioavailability of minerals and trace elements and can form calcium oxalate stones in the kidneys. Total oxalate content in quinoa ranged from 143 to 232 mg 100 g^−1^ in roots and grains, and from 874 to 1959 mg/100 g^−1^ in leaves and stems of quinoa [81]. Leaves of *C. album* contained oxalic acid ranged from 360 to 2000 mg 100 g^−1^ DW [82,83].

### 4.4. Trypsin Inhibitor (TI)

Trypsin inhibitor interacts with enzymes and makes them unavailable for protein digestion. TI is a thermolabile compound and is inactivated by heat treatment. To date, no reports are available on the detection of TI in quinoa leaves. However, Sood et al. (2012) [80] detected a very small amount of TI in *C. album* leaves, ranging from 0.11 to 0.17 TIU mg^−1^. TI contents in quinoa grains range from 1.36 to 5.04 TIU/mg which is much lower than in soybean (24.5 TIU mg^−1^) [84].

### 4.5. Tannins

Tannins are polyphenolic and antinutritional components and are able to interact with proteins and macromolecules, thus decreasing the nutritional value of food [63]. They are present in quinoa grains in small amounts, with values ranging from 23.00 to 31.00 mg 100 g^−1^ [47]. So far, there are no data available on the presence of tannins in quinoa greens.

## 5. Conclusions

Quinoa greens (green leaves, sprouts, and microgreens) are excellent sources of nutrients and health promoting compounds with antimicrobial, anticancer, antidiabetic, antioxidant, antiobesity, and cardio-beneficial properties. Greens are gluten-free and provide an outstanding source of protein, amino acids, essential minerals, and omega-3 fatty acids. Quinoa greens represent a promising value-added new vegetable that could resolve malnutrition problems and contribute to food and nutritional security. However, consumption of quinoa greens as vegetables is not common today. Greens can be eaten as both cooked vegetables and salads. In addition, quinoa greens may be combined as functional food ingredients in other gluten-free food products. The dried leaf powder may be added to soups, breads, and processed foods. Higher levels of total phenolic content and antioxidant capacity are found in germinated quinoa (quinoa sprouts) when compared to grains. After germination (sprouting), antioxidant capacity of quinoa sprouts increases for up to 6–9 days, and the amount of antioxidant is higher in red quinoa than yellow quinoa. Just like the sprouts and microgreens found in the stores today, quinoa sprouts and microgreens present a promising food for nutrition and human health. Further investigation is needed to draw a complete picture of the nutritional and functional significance to human health and to bring awareness to the use of quinoa greens in human diets as new ”super vegetables”.

## Figures and Tables

**Table 1:** Recent studies on nutritional composition and bioactive components in quinoa (*Chenopodium quinoa*) plant parts. Numbers in parenthesis refer to citations of articles of recent studies on quinoa plant parts.

Plant Parts	Study Description	Study Location	References
Leaf	Antioxidant and anticancer activities	Poland	[7]
Leaf	Nutritional and chemical composition	Egypt	[10]
Leaf	Nutritional parameters	Poland	[11]
Leaf	Nutritional contents	USA	[12]
Leaf	Proximate and chemical composition	Peru	[22]
Leaf	Antioxidant activity and nitric oxide production	Taiwan	[23]
Leaf	Nutritional composition and antioxidant capacity	Poland	[24]
Leaf	Nutritional and chemical composition	Egypt	[25]
Leaf	Nutritional composition, polyphenols, flavonoids, and antioxidant capacity	Mexico	[26]
Leaf and sprout	Phenolic composition and antioxidant capacity in colored seeds	Poland	[27]
Seed and plant	Fatty acid and nutritive value of quinoa grains and plants at different growth stages	Italy	[28]
Sprout	Nutritional and functional contents in sprouts	China	[29]
Sprout	Effect of processing on nutritional composition	India	[30]
Sprout	Phenolic contents in colored grains	Peru	[31]
Sprout	Phenolic composition and antioxidant capacity	Argentina	[32]
Sprout	Polyphenolic contents and antioxidant capacity	Ireland	[33]
Sprout	Phenolic profiles and antioxidant capacity	Saudi Arabia	[34]
Sprout	Polyphenols and antioxidant capacity	Poland	[35,36]
Sprout	Polyphenols and antioxidant capacity	China	[37]
Infructescence	Nutritional composition and antioxidant capacity	Poland	[24]
Inflorescence	Anticancer, antimicrobial, and antioxidant compounds	Pakistan	[38]

**Table 2:** Nutritional composition of quinoa plant parts and *C. album* leaves. Numbers in parenthesis refer to citations from which the nutrients data for quinoa plant parts and *C. album* were obtained.

Nutrients	Quinoa Plant Parts			*C. album* Leaves
	Leaves	Sprouts	Grains	
Proximate composition				
Crude protein%	28.2–37.0 [10,12]	6.1–12.3 [30,39]	9.1–15.7 [40]	28.7 [13]
Crude fat%	2.4–4.5 [10,12]	0.1–3.8 [30,39]	4.0–7.6 [40]	4.4 [13]
Crude fiber%	6.9–7.8 [10,12]	4.6–23.5 [30,39]	7.0–14.1 [40,41]	0.1 [13]
Carbohydrate%	34.0 [12]	9.6–73.0 [30,39]	48.5–69.8 [40]	40.8 [13]
Ash%	2.1–20.0 [10,12]	0.9–3.4 [30,39]	2.0–7.7 [40]	21.0 [13]
Energy (kcal)	325 [12]	69 [39]	331–381 [40]	317.8 [13]
Essential amino acids (g 100 g^−1^ DW)				
Histidine (His)	0.7 [12]	0.7 [29]	1.4–5.4 [40]	0.4 [42]
Isoleucine (Ile)	1.6 [12]	1.1 [29]	0.8–7.4 [40]	0.5 [42]
Leucine (Leu)	2.7 [12]	2.0 [29]	2.3–9.4 [40]	1.3 [42]
Lysine (Lys)	1.9 [12]	1.3 [29]	2.4–7.5 [40]	1.8 [42]
Methionine (Met)	0.6 [12]	0.2 [29]	0.3–9.1 [40]	0.2 [42]
Phenylalanine (Phe)	1.8 [12]	1.2 [29]	0.1–2.7 [40]	0.9 [42]
Threonine (Thr)	1.5 [12]	1.0 [29]	2.1–8.9 [40]	0.8 [42]
Tryptophan (Trp)	1.2 [12]	NA	0.6–1.9 [40]	NA
Valine (Val)	1.8 [12]	1.3 [29]	0.8–6.1 [40]	0.7 [42]
Minerals (mg 100 g^−1^ DW)				
Calcium (Ca)	147.0–1535.0 [10,12]	21.7 [29]	27.5–148.7 [40]	1438.9 [13]
Copper (Cu)	1.0–1.1 [10,12]	0.2 [29]	1.0–9.5 [40]	1.1 [13]
Iron (Fe)	11.6–148.0 [10,12]	NA	1.4–16.7 [40]	15.2 [13]
Magnesium (Mg)	14.0–902.0 [12,43]	219.3 [29]	26.0–502.0 [40]	1301.1 [13]
Phosphorus (P)	39.0–405.6 [12,43]	NA	140.0–530.0 [40]	419.7 [13]
Potassium (K)	474.0–8769.0 [12,43]	525.2 [29]	696.7–1475.0 [40]	8125.2 [13]
Sodium (Na)	3.0–15.1 [10,12]	NA	11.0–31.0 [40]	573.9 [13]
Zinc (Zn)	3.3–6.8 [10,12]	NA	2.8–4.8 [40]	4.8 [13]

DW: dry weight; NA, not available.

**Table 3:** Fatty acid profile of quinoa plant parts at different growth stages, spinach, and kale. Numbers in parenthesis refer to citations from which the fatty acid profile data were obtained.

Plant Parts Studied	Saturated Fatty Acid (SFA)		Unsaturated Fatty Acid (UFA)			References
	Palmitic (16:0)	Stearic (18:0)	Oleic (18:1)	Linoleic Acid (LA, 18:2)	Linolenic Acid (ALA, 18:3)	
Quinoa early veg	12.07	1.51	7.49	15.97	47.4	[28]
Quinoa bud	11.64	1.68	7.64	16.14	39.9	[28]
Quinoa grain	9.60–10.00 [9]	0.84–0.94 [9]	23.10–29.18 [9,47]	46.69–58.10 [9,47]	6.10–8.44 [9,47]	[9,47]
Spinach	20.65	1.71	9.48	18.63	37.37	[48]
Kale	11.84	3.95	2.14	11.8	54	[49]

**Table 4:** Total phenolic content (TPC), total flavonoid content (TFC), and antioxidant capacity (AC, measured using 2,2-diphenyl-1-picrylhydrazyl-DPPH assay) in quinoa plant parts. Numbers in parenthesis refer to citations from which the TPC, TFC, and AC data were obtained.

Plant Parts Studied	TPC	TFC	AC_DPPH	References
	(mg GAE 100 g^−1^ DW)	(mg QE 100 g^−1^ DW)	(mg TE 100 g^−1^ DW)	
Leaf	418.00–544.00	14.00–23.00	29.90–55.40	[10]
Leaf	10.55–10.75 mg/g	8.69–9.14 mg/g	46.00–62.65	[22]
Leaf	569.50 mg/g	NA	50.70–65.30 mg/g	[23]
Leaf	188	NA	34	[24]
Leaf	131.8	62.07	NA	[26]
^1^ Leaf	16.03–16.10 mg/g	2.02–2.54 mg/g	NA	[27]
Sprout	58.91–71.01 mg/g	3.29–9.05 mg/g	NA	[29]
Sprout	101.2	18.02	61.41	[61]
^1^ Sprout	308.82–417.75	NA	NA	[31]
Sprout	79.04	NA	27.39	[32]
Sprout	147	122.6	50.4	[33]
^1^ Sprout	259.02–293.35	10.38–24.00	5.26–7.39	[34]
Sprout	49.02–51.63	290.00–304.10	NA	[35,36]
^1^ Sprout	15.15–28.79 mg/g	0.63–3.34 mg/g	NA	[27]
Infructescence	172	NA	95	[24]
Quinoa grain, raw	39.29–198.23 [31,32]	11.40–223.80 [36,61]	13.61–59.61 [33,61]	

NA, not available; ^1^ Colored grain.

## Data Availability

Not applicable.
